# 

**Published:** 1906-04-14

**Authors:** 


					the hospital. lu? Apbil 14th,1906
TALr
Lt
as cow Western Infirmary ' ? " ^floMojJiAMBJvuRty/CH tiOSMt&ub
NO. 1,020. Vol. XL. [af^pfper!] ? SATURDAY,
1906.
[Subscription^Terms] pR|CE -|-||||??p?||Qg

				

